# Diagnostic Performance of Saline Infusion Sonography with Color Doppler Flow (SIS-CF) for Assessing Tubal Patency: A Prospective Cohort Study

**DOI:** 10.3390/diagnostics16091287

**Published:** 2026-04-24

**Authors:** Pantana Bangsomboon, Worashorn Lattiwongsakorn, Tawiwan Panthasri, Ubol Saeng-anan, Usanee Sanmee, Natnita Mattawanon, Natpat Jansaka

**Affiliations:** Division of Reproductive Medicine, Department of Obstetrics and Gynaecology, Faculty of Medicine, Chiang Mai University, Chiang Mai 50200, Thailand; panoey.b@gmail.com (P.B.);

**Keywords:** color Doppler flow, chromopertubation, laparoscope, saline infusion sonography, tubal patency

## Abstract

**Background/Objectives**: Accurate evaluation of fallopian tube patency is an essential step in infertility assessment. This study investigated the diagnostic capability of saline infusion sonography combined with color Doppler flow (SIS-CF), using laparoscopic chromopertubation as the comparator method. **Methods**: A prospective diagnostic accuracy study was conducted between January and November 2025 at the Infertility Unit, Chiang Mai University Hospital, Thailand. Women requiring laparoscopic assessment for infertility evaluation or preconception investigation were consecutively enrolled. Each participant underwent SIS-CF immediately before laparoscopic chromopertubation within the same operative session. Primary outcomes included diagnostic indices for tubal patency. Secondary outcomes included procedural duration and perioperative safety. **Results**: Forty-four women (88 fallopian tubes) were included. SIS-CF demonstrated sensitivity of 89.4% (95% CI 78.1–95.9) and specificity of 95.5% (95% CI 77.2–99.9). Positive and negative predictive values were 98.3% and 75.0%, respectively. Likelihood ratios were 19.65 (positive) and 0.11 (negative), with overall accuracy of 90.9% (95% CI 82.9–95.8). Median procedure duration was 5.7 min for SIS-CF and 3.0 min for laparoscopic chromopertubation. No adverse events were recorded. **Conclusions:** SIS-CF demonstrated favorable diagnostic characteristics for evaluation of tubal patency. The technique provided functional information regarding tubal status while maintaining a minimally invasive profile. Further investigation is warranted to determine its role within routine infertility workups.

## 1. Introduction

Infertility represents an escalating challenge to global health, with rising rates observed in both industrialized and developing nations. Current estimates suggest that infertility impacts approximately 5–8% of populations in high-income countries and between 3.5% and 16.7% in lower-resource regions [1]. Thailand has reported rates reaching as high as 51.5% [2]. The causes of infertility are heterogeneous and may involve both male and female components; however, abnormalities affecting the fallopian tubes contribute significantly, accounting for approximately one-quarter to two-fifths of cases [3,4,5]. Determination of tubal patency is therefore a fundamental component of infertility evaluation. According to the European Society of Human Reproduction and Embryology (ESHRE) [6], laparoscopic chromopertubation is regarded as the reference approach because it allows direct confirmation of dye passage through the fimbrial ends of the tubes. Nevertheless, its high costs, invasive nature, and associated surgical risks often preclude its routine clinical use.

Various diagnostic techniques have emerged to assess tubal status. Of them, hysterosalpingography (HSG) is widely utilized as a primary screen, but its reliance on iodinated contrast agents can lead to allergic reactions or pelvic infections [7]. Ultrasound-based modalities, including hysterosalpingo-contrast sonography (HyCoSy) and hysterosalpingo-foam sonography (HyFoSy), have demonstrated favorable diagnostic performance [8]. However, the necessary contrast media are frequently unavailable in many healthcare settings, including Thailand. Furthermore, meta-analyses suggest contrast intravasation occurs in approximately 0.6% of HyCoSy/HyFoSy examinations, although higher rates—up to 23%—have been reported in studies utilizing SonoVue^®^ [9].

SIS shows relatively high sensitivity (approximately 84–100%) and specificity (66–97%), often comparable or superior to HSG and approaching the diagnostic accuracy of laparoscopy [5,10,11,12,13,14,15,16]. However, reported diagnostic accuracy varies considerably among studies, depending on the type of contrast agent used, the quality of ultrasound imaging, and operator expertise. Because of this variability in reported accuracy, further improvement remains necessary, and several modifications have been proposed to enhance diagnostic performance. Recent advances in ultrasound technology, particularly spatial resolution and enhanced color Doppler flow, enable better visualization of low-velocity fluid movement. We hypothesized that integrating color Doppler flow into SIS (SIS-CF) would enhance visualization of saline flow through the fallopian tubes, thereby refining diagnostic accuracy. This approach offers a safe, minimally invasive, and cost-effective alternative that is particularly suitable for low-resource environments since it eliminates the need for specialized contrast agents. To the best of our knowledge, reports of SIS-CF utilizing contemporary transvaginal systems are scarce in the literature. Therefore, the primary aim of this study was to evaluate the diagnostic accuracy of SIS-CF for assessment of tubal patency using laparoscopic chromopertubation as the reference standard. Secondary objectives included comparing procedural durations, evaluating safety and complications, and determining the method’s effectiveness in detecting concurrent pelvic pathology.

## 2. Materials and Methods

### 2.1. Study Design and Participants

This prospective diagnostic study was conducted at Maharaj Nakorn Chiang Mai Hospital, Chiang Mai University, Thailand, a tertiary referral center and medical school, between January and November 2025. The research was designed to evaluate the diagnostic efficacy of saline infusion sonography with color Doppler flow (SIS-CF) in assessing fallopian tube patency, employing laparoscopic chromopertubation as the reference standard.

Women undergoing laparoscopic evaluation as part of infertility investigation or management were consecutively recruited. The inclusion criteria were as follows: (1) women aged 20–45 years and (2) those undergoing infertility evaluation or preconception assessment. The exclusion criteria were as follows: (1) active pelvic infection, (2) pregnancy, and (3) known hypersensitivity to saline or contrast materials. All participants underwent SIS-CF followed by laparoscopic chromopertubation during the same operative session.

### 2.2. Ethical Considerations and Informed Consent

Eligible candidates were admitted to the hospital prior to surgery according to standard institutional practice. On the day of their admission, all subjects received detailed verbal and written information regarding the study procedures. This briefing covered the study’s objectives, the SIS-CF technique, anticipated outcomes, and potential advantages for clinical decision-making in infertility care. It was clearly stated that participation was entirely voluntary and that SIS-CF would be performed just prior to the reference laparoscopic procedure.

Potential risks—including uterine cramping, infections, allergic reactions, and other complications related to the procedure—were thoroughly discussed with each participant. Before enrollment, every participant provided written informed consent. They were informed that choosing not to participate would not interfere with their standard medical treatment.

The ethics committee at the Faculty of Medicine, Chiang Mai University, approved the study protocol (Study ID: OBG-2567-0594). Candidates who met all criteria were consecutively enrolled and scheduled for the procedures in a sequential order.

### 2.3. The Procedure

SIS-CF procedure: After the induction of general anesthesia, participants were positioned in the dorsal lithotomy position. The SIS-CF examination was conducted by the first team consisting of a reproductive medicine fellow and a gynecologic resident, both of whom possessed specialized training in saline infusion and transvaginal ultrasound techniques. A sterile vaginal speculum was inserted. A No. 8 pediatric Foley catheter was introduced into the uterine cavity, and the balloon was inflated with 5 mL of normal saline solution (NSS) to prevent catheter displacement. Thereafter, 0.9% NSS was slowly infused to distend the uterine cavity and allow passage through both fallopian tubes. The procedure was performed under simultaneous transvaginal ultrasonographic guidance using a GE Voluson P6 ultrasound system. (GE Healthcare, Milwaukee, WI, USA) equipped with transabdominal 2–4 MHz and transvaginal 5–7 MHz transducers. During SIS-CF, the color Doppler box was centered over each fallopian tube using low-flow parameters and a pulse repetition frequency (PRF) of <1 kHz. Color Doppler mode was activated to assess tubal flow. Each fallopian tube was evaluated separately by identifying the expected anatomical course of the tube and assessing the passage of saline under real-time ultrasound visualization. Tubal patency was indicated by visualization of color Doppler flow (blue signals moving away from the probe) and the accumulation of free fluid around the ovaries. In addition to tubal evaluation, the uterus, endometrium, ovaries, and adnexal structures were evaluated for structural abnormalities, including endometrioma, dermoid cyst, hydrosalpinx, uterine anomaly, myoma, endometrial polyp, and adenomyosis. Endometrial thickness and uterine morphology were also assessed as part of routine transvaginal ultrasonographic examination. After completion of SIS-CF, the Foley catheter was removed.

To ensure high reproducibility and minimize operator-dependent bias, all SIS-CF procedures adhered to a standardized institutional protocol. Participants were continuously monitored for any allergic reactions or procedural complications throughout the session.

Laparoscopic chromopertubation (reference standard): Following SIS-CF, the first team exited the operating room and laparoscopic chromopertubation was performed by a second independent surgical team—comprising a reproductive medicine fellow and an attending gynecologic staff. The second team entered the operating room only after the SIS-CF procedure had been completed. Laparoscopic chromopertubation was performed immediately after SIS-CF during the same operative session under general anesthesia. A Leech–Wilkinson cannula was inserted through the cervix for the intrauterine injection of indigo-carmine dye. Tubal patency was confirmed by laparoscopic visualization of dye spillage from the fimbrial ends of the fallopian tubes. The laparoscopic team did not participate in the SIS-CF procedure or interpretation. Although complete blinding could not be definitively guaranteed due to the intraoperative setting, the use of separate teams performing the procedures sequentially was intended to reduce observer bias.

Pelvic findings including endometriosis, endometrioma, dermoid cyst, hydrosalpinx, uterine anomaly, myoma, endometrial polyp, adenomyosis, parovarian cyst, and pelvic adhesions were documented based on these laparoscopic findings.

### 2.4. Definitions and Outcome Measures

The primary outcome measure was tubal patency for each fallopian tube, as assessed by both procedures. Secondary endpoints included the time required to complete each procedure and the occurrence of laparoscopic complications.

Tubal patency on SIS-CF was defined as visualization of saline movement along the anticipated course of the fallopian tube, demonstrated by color Doppler flow signals. Patency on laparoscopy was confirmed by direct visualization of dye efflux from the fimbrial end following transcervical injection.

Baseline variables comprised age, body mass index (BMI), infertility categories (primary or secondary), duration of infertility, previous diagnosis of endometriosis or endometrioma, prior pelvic inflammatory disease (PID), and history of abdominal or pelvic surgery. Historical variables referred to medical conditions known before enrollment.

For evaluation of pelvic abnormalities, each pathology was evaluated separately; therefore, participants with more than one condition were included across multiple diagnostic categories.

Participants were observed for any procedure-associated adverse events intraoperatively and during the postoperative hospital stay. Events of interest included infection, allergic reaction, uterine injury, abnormal bleeding, or other adverse events related to SIS-CF. Postoperative follow-up was maintained until hospital discharge, which typically occurred within 2 days after surgery according to institutional practice. No additional follow-up was performed because the primary objective of the study was diagnostic accuracy assessment.

**Figure 1:**
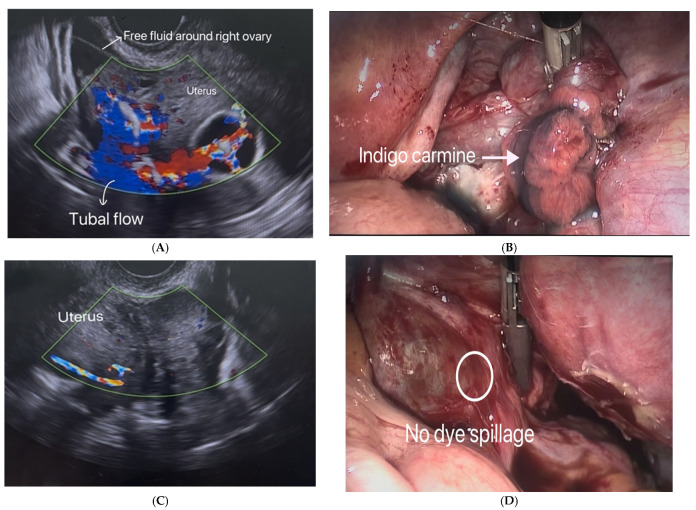
(**A**) Tubal patency of the right fallopian tube on SIS-CF: blue color Doppler signals indicating saline flow from the endometrial cavity through the fallopian tube into the pelvic cavity, with free fluid around the right ovary. (**B**) Tubal patency of the right fallopian tube on laparoscopic chromopertubation: visualization of indigo carmine dye passing through the fallopian tube into the pelvic cavity. (**C**) Occlusion of the left fallopian tube on SIS-CF: no flow observed from the endometrial cavity through the fallopian tube into the pelvic cavity and no free fluid around the ovary. (**D**) Occlusion of the left fallopian tube on laparoscopic chromopertubation: no visualization of indigo carmine dye within the pelvic cavity.

Examples: of SIS-CF and laparoscopic chromopertubation images demonstrating tubal patency and occlusion are illustrated in Figure 1.

### 2.5. Statistical Analysis

Data processing and analysis were conducted using IBM Statistical Package for the Social Sciences (SPSS) Statistics for Windows, version 26.0 (2019; IBM Corp., Armonk, NY, USA). Demographic characteristics are summarized as number (percentage), mean ± standard deviation, or median (interquartile range), as appropriate. Normality of continuous variables was assessed using the Shapiro–Wilk test. Normally distributed data are reported as mean ± standard deviation, whereas non-normally distributed data are expressed as median (interquartile range: IQR). To evaluate the diagnostic performance of SIS-CF in assessing tubal patency, with laparoscopic chromopertubation as the comparator technique, sensitivity, specificity, positive and negative predictive values, likelihood ratios, and overall accuracy were calculated. The level of concordance between SIS-CF and laparoscopic results was quantified using Cohen’s kappa coefficient. We defined the detection rate as the percentage of fallopian tubes for which SIS-CF provided a conclusive assessment of patency. Procedure durations were compared using the Wilcoxon signed-rank test, with statistical significance established at a *p*-value < 0.05. Receiver Operating Characteristic (ROC) curve analysis was performed to determine the area under the curve (AUC) with 95% confidence intervals. Because SIS-CF yields dichotomous outcomes, ROC analysis was included as a secondary descriptive measure of discrimination, while conventional diagnostic indices remained the primary basis for interpretation.

The sample size was calculated to evaluate the diagnostic accuracy of SIS-CF in identifying tubal occlusion. Based on institutional data from 2023 indicating a 20.9% prevalence, the calculation assumed an expected sensitivity of 95%, a significance level (α) of 0.05, and 10% precision. It was determined that a minimum of 87 fallopian tubes was necessary, which required the recruitment of 44 participants (assuming two tubes per woman).

## 3. Results

### 3.1. Recruitment Flow and Baseline Characteristics

**Figure 2:**
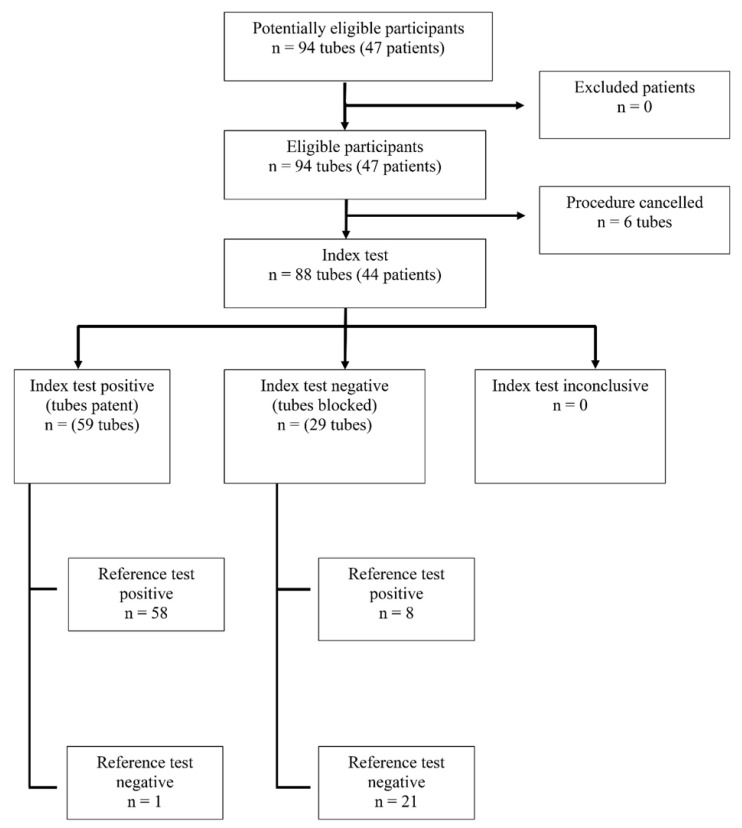
The flowchart of patient recruitment.

During the recruitment period, 50 women were screened for study eligibility. Six individuals declined participation, leaving 44 women (88 fallopian tubes) included in the final dataset (Figure 2). Baseline demographic characteristics and pre-existing pelvic pathologies identified during clinical evaluation are summarized in Table 1. The mean age was 32.75 years, with a mean body mass index of 22.50 kg/m^2^. Duration of infertility showed a non-normal distribution and was described using the median (range), with a median duration of 4.5 years. More than half of participants (59%) had primary infertility. A prior diagnosis of endometriosis or endometrioma was reported in 34.1% of women, while 15.9% had undergone previous abdominal or pelvic surgery. A history of pelvic inflammatory disease was documented in 6.8% of cases. The most frequent underlying condition identified before enrollment was endometriosis, accounting for more than 40% of cases, including simple endometriotic lesions (approximately 17%) and endometriomas (nearly 25%). Other associated lesions included dermoid cysts (15.38%) and hydrosalpinx (10.77%).

### 3.2. Pelvic Pathology

Preoperative transvaginal ultrasonographic findings, together with corresponding laparoscopic and hysteroscopic diagnoses, are summarized in Table 2. For analytical purposes, each pathological condition was considered separately; therefore, participants presenting with more than one abnormality were counted in multiple diagnostic groups. Preoperative transvaginal ultrasonography demonstrated high concordance with laparoscopic and hysteroscopic findings for common pelvic pathologies. All cases of hydrosalpinx, endometrioma, and dermoid cysts detected by ultrasonography were subsequently confirmed during surgery. Likewise, all four endometrial polyps detected on ultrasonography were verified hysteroscopically, indicating complete concordance for endometrial polyp detection in this cohort. As anticipated, endometriotic implants without cystic components could not be identified on preoperative ultrasonography. Detailed diagnostic performance for each pelvic condition is presented in Table 2.

### 3.3. Diagnostic Performance of SIS-CF

**Figure 3:**
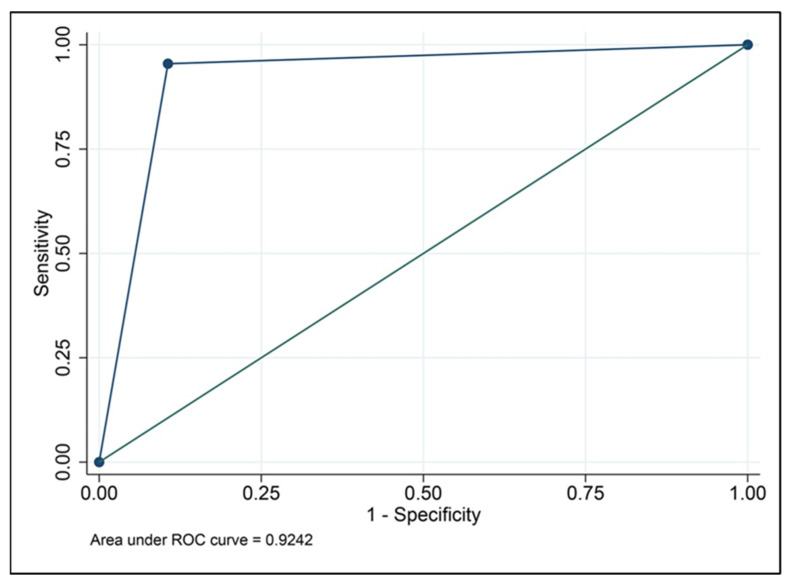
The receiver operating characteristic (ROC) curve of the diagnostic performance of SIS-CF in predicting tubal occlusion.

All 88 fallopian tubes were successfully evaluated by both SIS-CF and laparoscopic chromopertubation. The prevalence of tubal patency in this cohort was 75% (66 of 88 fallopian tubes). The contingency analysis of SIS-CF compared with laparoscopic chromopertubation is presented in Table 3. SIS-CF correctly identified tubal patency in 59 of 66 patent tubes and correctly identified tubal occlusion in 21 of 22 occluded tubes. The diagnostic performance of SIS-CF in predicting tubal patency demonstrated a sensitivity of 89.4% (95% CI 79.4–95.6) and a specificity of 95.5% (95% CI 77.2–99.9) (false-positive rate, 4.5%), as presented in Table 3. The overall diagnostic accuracy was 90.9% (95% CI, 82.9–95.8%). The positive likelihood ratio was 19.67, indicating a marked increase in the likelihood of tubal patency when SIS-CF demonstrated flow. The negative likelihood ratio was 0.11, suggesting a marked reduction in probability when SIS-CF indicated tubal occlusion. Agreement between SIS-CF and laparoscopic chromopertubation was substantial, with a Cohen’s kappa coefficient of 0.78. The area under the receiver operating characteristic (ROC) curve was 0.92 (95% CI 0.87–0.98), indicating good overall discrimination. ROC results are presented as a complementary measure alongside conventional diagnostic performance indices, as presented in Figure 3.

Analyses were conducted at the tubal level, with each fallopian tube treated as an independent unit of analysis. False-negative SIS-CF (false occlusion) results (*n* = 7) were primarily attributable to anatomical or technical factors. Two cases occurred in women with a septate uterus, in whom blind intrauterine catheter placement likely impeded saline flow into the contralateral uterine cavity. The remaining false-negative results were observed in women with large endometriomas, which may have impaired visualization or restricted saline passage through the fallopian tube. Overall, false-negative findings were more frequently associated with uterine anomalies and large ovarian endometriomas.

Only one false-positive (false patency) result was identified. This discrepancy may be explained by minimal dye passage during laparoscopic chromopertubation, which was insufficient to generate detectable color Doppler flow on SIS-CF.

### 3.4. Procedure Duration

**Figure 4:**
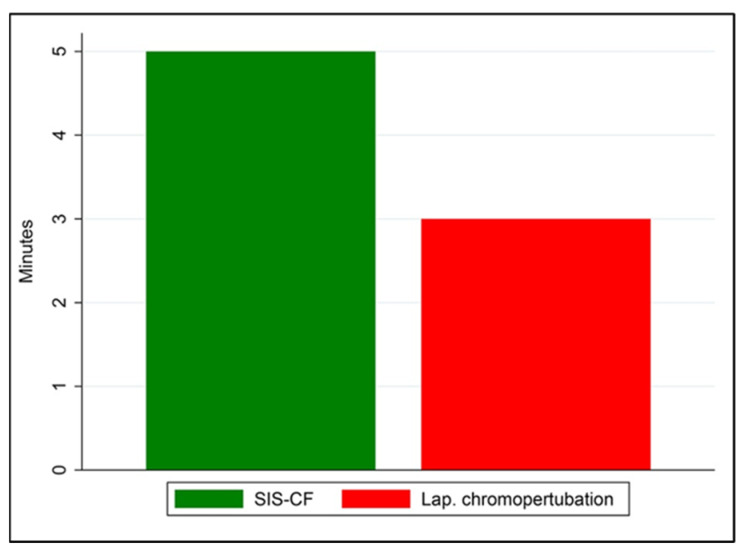
Compare the duration between SIS-CF and laparoscopic chromopertubation.

The median procedure duration of SIS-CF was significantly longer than that of laparoscopic chromopertubation, at 5.00 min (range, 5–10 min) compared with 3.00 min (range, 2–5 min) (Wilcoxon signed-rank test; *p* < 0.001), as illustrated in Figure 4. Although the measured procedural duration of laparoscopic chromopertubation was shorter, this comparison reflects only the chromopertubation step and does not account for anesthesia induction, surgical preparation, or recovery time.

### 3.5. Complications and Safety

No immediate intraoperative or early postoperative adverse events were observed until discharge. However, these findings should be interpreted with caution, as the study was not designed to assess patient-reported outcomes such as tolerability, pain, or procedural convenience in a first-line outpatient setting.

## 4. Discussion

This investigation assessed the performance of Doppler-assisted saline infusion sonography for evaluation of fallopian tube patency in comparison with laparoscopic chromopertubation. Visualization of fluid movement using color Doppler imaging enabled functional assessment of tubal flow dynamics during the procedure. The observed results indicate that SIS-CF can discriminate between patent and occluded tubes with a high level of agreement relative to the surgical reference method when performed using a standardized protocol.

The diagnostic indices observed in this study demonstrated favorable performance characteristics. Sensitivity approached 90% and specificity exceeded 95%, with overall accuracy above 90%. The area under the ROC curve indicated strong discriminative ability, and agreement analysis demonstrated substantial concordance between the two diagnostic approaches. However, given the dichotomous nature of tubal patency assessment, ROC findings should be interpreted as supportive rather than primary evidence of diagnostic performance. These findings fall within ranges previously reported for ultrasound-based methods for tubal patency evaluation and are comparable to values described for hysterosalpingography [17]. Collectively, the results suggest that the addition of Doppler flow assessment to saline infusion sonography may provide clinically meaningful functional information regarding tubal status.

Because the present study did not include a direct comparison between conventional saline infusion sonography and Doppler-enhanced assessment, the specific incremental value attributable to Doppler imaging cannot be determined. Accordingly, the reported findings should be interpreted as reflecting the diagnostic performance of the integrated SIS-CF approach rather than the independent effect of Doppler flow visualization alone. Differences in diagnostic performance reported across previous studies may reflect variation in equipment, technique, and operator experience [5,10,11,12,13,14,15,16]. The concordance rate observed in this cohort (90.91%) is comparable to the 86.7% agreement reported by Exacoustos et al. [17]. In addition, the diagnostic indices in this study are similar to values previously reported for hysterosalpingography [10]. These findings suggest that incorporation of Doppler assessment into saline infusion sonography may provide a reliable alternative approach for evaluating tubal patency. Emerging techniques capable of detecting very low-velocity flow signals, such as superb microvascular imaging [18], may further improve visualization of saline movement and enhance interpretation of flow patterns.

Only one false-positive result was observed, likely reflecting technical or interpretive variation. False-negative results were frequently associated with anatomical or procedural factors. Two cases occurred in women with a septate uterus, in whom blind Foley catheter placement into a single cavity may have prevented saline flow to the contralateral tube, leading to apparent occlusion. This highlights the need for anatomical awareness when performing the procedure in patients with Müllerian anomalies. The remaining five false-negative results were observed in women with large endometriomas, which may distort adnexal anatomy or interfere with visualization of fluid passage.

Beyond tubal assessment, conventional transvaginal ultrasonography (TVUS) with saline infusion can provide valuable structural information. Saline instillation facilitates delineation of the endometrial cavity and identification of intracavitary abnormalities such as endometrial polyps, synechiae, uterine septa or lesions suspicious for malignancy, thereby facilitating appropriate further evaluation, including MRI when clinically indicated [19]. Characterization of adnexal pathology primarily relies on grayscale ultrasonography. Prior evidence supports the diagnostic value of ultrasound-based techniques in this context. Yazbek et al. [20] demonstrated high diagnostic performance of transvaginal ultrasound (TVUS) for benign adnexal masses compared with laparoscopy, while multiple studies [21,22,23,24] have shown that saline infusion sonography (SIS) improves detection of intracavitary pathology relative to conventional TVUS and provides diagnostic performance comparable to hysteroscopy. Importantly, the color Doppler flow component is intended mainly to support tubal patency assessment through flow-related signs; the incremental contribution of color Doppler for the detection of pelvic pathology beyond TVUS/SIS was not specifically quantified in our study. No procedure-related complications were observed in our cohort.

Several methodological considerations should be acknowledged. First, patient discomfort could not be evaluated because all procedures were performed under general anesthesia. Second, the relatively limited sample size and single-center setting may reduce external validity. Third, diagnostic accuracy was evaluated at the tubal level, with each fallopian tube treated as an individual observation. However, both tubes originate from the same patient and may share biological and clinical characteristics. As a result, observations may not be fully independent, which could lead to underestimation of variability and relatively narrow confidence intervals. Future studies applying statistical approaches that account for within-patient correlation may provide more precise estimates. Finally, the study population consisted of women selected for surgical evaluation, which may differ from the general infertility population encountered in outpatient practice. Predictive values observed in this cohort may therefore not directly reflect performance in lower-risk populations. Therefore, the present findings do not support the readiness of this technique for routine use as a real first-line outpatient test based on the presented data. Future investigations directly comparing standard saline infusion sonography with Doppler-assisted techniques may help clarify the independent contribution of Doppler flow assessment to overall diagnostic performance.

From a clinical perspective, SIS-CF may provide useful information during infertility evaluation. Demonstration of bilateral tubal patency may allow progression to ovulation induction or intrauterine insemination, whereas suspected bilateral occlusion may support earlier consideration of assisted reproductive techniques. Although superiority over conventional SIS has not yet been established, incorporation of color Doppler imaging allows visualization of fluid movement along the expected course of the fallopian tube, thereby providing additional functional information regarding tubal status. However, further comparative studies are required to clarify whether Doppler imaging independently improves diagnostic accuracy beyond conventional saline infusion sonography. In addition, saline infusion techniques have been reported to improve detection of intrauterine abnormalities [21,23,24]. Therefore, SIS-CF may provide complementary functional and structural information relevant to clinical decision-making.

Based on the findings of this study, further research is required to compare standardized SIS protocols with Doppler-enhanced approaches. Future studies should focus on establishing uniform procedural standards—including saline infusion techniques, Doppler settings, and operator training—to ensure reproducibility across multicenter clinical settings.

## 5. Conclusions

Integration of color Doppler flow assessment into saline infusion sonography demonstrated encouraging diagnostic characteristics for the identification of tubal patency. The approach provides real-time functional information without requiring specialized contrast agents and may be applicable in settings with limited access to alternative diagnostic resources. Because this study was conducted in a tertiary referral population undergoing laparoscopy under general anesthesia, extrapolation of these findings to routine outpatient infertility evaluation should be interpreted with caution. Additional investigations are necessary to determine reproducibility and to clarify the role of this technique within standardized infertility evaluation pathways.

## Figures and Tables

**Table 1:** Baseline characteristics of the patients.

Variable	Value (*n* = 44)
Age (years), mean ± SD (range)	32.75 + 4.94 (20–44)
BMI (kg/m^2^), mean ± SD (range)	22.50 + 4.18 (17.36–35.00)
Infertility type Primary infertility, *n* (%) Secondary infertility, *n* (%)	26 (59%) 18 (40.9%)
Duration of infertility (years), median (range)	4.5 (1–13)
History of endometriosis or endometrioma *, *n* (%)	15 (34.1)
Previous abdominal or pelvic surgery *, *n* (%)	7 (15.9)
History of pelvic inflammatory disease (PID) *, *n* (%)	3 (6.8)

Values are presented as mean ± standard deviation, median (range), or number (percentage), as appropriate. * History refers to conditions known prior to study enrollment. Previous abdominal or pelvic surgery includes appendectomy, cesarean section, ovarian surgery, or other abdominal procedures. Baseline characteristics are reported at the patient level (*n* = 44).

**Table 2:** Pelvic pathology detected by ultrasonography compared with laparoscopic/hysteroscopic diagnosis.

Laparoscopic and Hysteroscopic Diagnosis	Confirmed by Reference Standard *n* (%)	Ultrasonography Positive *n*	Detected Among Confirmed (TP/Confirmed), *n*/*N* (%)
Dermoid cyst	10 (15.38)	9	9/10 (90.00)
Endometrioma	16 (24.62)	14	14/16 (87.50)
Endometriosis	11 (16.92)	0	0/11 (0.00)
Ovarian/parovarian cysts	5 (6.13)	6	5/5 (100.00)
Anterior abdominal wall adhesion	1 (1.53)	0	0/1 (0.00)
Adenomyosis	1 (1.53)	2	1/1 (100.00)
Myoma	7 (10.77)	8	7/7 (100.00)
Bicornuate/septate uterus	2 (3.07)	2	2/2 (100.00)
Endometrial polyp	6 (9.23)	4	4/6 (66.67)
Hydrosalpinx	7 (10.77)	5	5/7 (71.43)

Ultrasonography positive includes true-positive and false-positive findings. Detection among confirmed cases was calculated as true positives divided by surgically confirmed cases (TP/Confirmed).

**Table 3:** Diagnostic performance of SIS-CF in predicting tubal patency, using laparoscopic chromopertubation as the reference standard.

	Occlusion by SIS-CF		
Final Diagnosis (Lap-CT)	Negative Test	Positive Test	Total
Occlusion	21	1	22
Patent	7	59	66
Total	28	60	88
Diagnostic indices	Values	95% CI (lower)	95% CI (upper)
Prevalence	75.00%	64.60%	83.60%
Sensitivity	89.40%	79.40%	95.60%
Specificity	95.50%	77.20%	99.90%
ROC area	0.92	0.87	0.98
Positive predictive value	98.30%	91.10%	100.00%
Negative predictive value	75.00%	55.10%	89.30%
Positive likelihood ratio	19.67	2.89	133.7
Negative likelihood ratio	0.11	0.05	0.23

## Data Availability

The datasets analyzed during the current study are available from the corresponding author upon reasonable request.

## Abbreviations

The following abbreviations are used in this manuscript:

SIS-CF	saline infusion sonography with color Doppler flow
HSG	hysterosalpingography
NSS	normal saline solution
SPSS	Statistical Package for the Social Sciences
